# Rearing Laying Hens in Aviaries Reduces Fearfulness following Transfer to Furnished Cages

**DOI:** 10.3389/fvets.2016.00013

**Published:** 2016-02-26

**Authors:** Margrethe Brantsæter, Fernanda M. Tahamtani, Randi O. Moe, Tone B. Hansen, Rachel Orritt, Christine Nicol, Andrew M. Janczak

**Affiliations:** ^1^Animal Welfare Research Group, Department of Production Animal Clinical Science, NMBU School of Veterinary Science, Oslo, Norway; ^2^Animalia, Norwegian Meat and Poultry Research Centre, Oslo, Norway; ^3^School of Psychology, University of Lincoln, Lincoln, UK; ^4^Division of Animal Health and Husbandry, University of Bristol, Bristol, UK

**Keywords:** laying hens, chicken, welfare, rearing, development, fearfulness, stress, fear

## Abstract

Appropriate rearing is essential for ensuring the welfare and productivity of laying hens. Early experience has the potential to affect the development of fearfulness. This study tested whether rearing in aviaries, as opposed to cages, reduces the fearfulness of laying hens after transfer to furnished cages. Fear responses were recorded as avoidance of a novel object in the home cage. Lohmann Selected Leghorns were reared in an aviary system or conventional rearing cages and then transported to furnished cages at 16 weeks, before the onset of lay. Observations of a selection of birds were conducted at 19 (*N* = 50 independent cages) and 21 (*N* = 48 independent cages) weeks of age. At 19 and 21 weeks, cage-reared birds showed higher levels of fearfulness indicated by spending more time away from the novel object compared to aviary-reared birds. These results suggest that rearing in an enriched aviary environment reduces fearfulness up to the fifth week after transfer to a new housing system, compared to rearing in cages.

## Introduction

Under natural conditions, fear normally functions to protect animals from dangerous situations, and thereby increases their chances of survival (1). However, under production conditions, exaggerated fear is a potent stressor associated with activation of the hypothalamic–pituitary–adrenocortical axis. Fear may have negative consequences for animal welfare and productivity if the fear responses are exaggerated, inappropriate, or expressed in a restrictive environment (2–5). Fearfulness is the predisposition of an individual to be easily frightened (1, 6) and is influenced by both genetic (7) and developmental factors.

The early environment may have a great impact on the development of fearfulness and associated activation of the hypothalamic–pituitary–adrenocortical axis in response to stressors (6, 8–11). Exposure to increased environmental complexity during rearing has been found to reduce fearfulness during adulthood in several species, including mice (12), pigs (13), and chickens (14). For laying hens, the housing system during rearing is a major source of environmental variability, illustrated by the large difference between the cage and aviary-rearing systems. However, few studies have tested for effects of the rearing system on later fearfulness in laying hens. A study comparing floor-housed adult birds reared on sand, straw, or wire from 0 to 4 weeks found that birds reared on wire were the most fearful, as indicated by longer durations of induced tonic immobility (14). A study comparing cage-housed adult birds reared in a floor or cage system found that floor-reared birds were more active, and displayed more flighty responses to a human than cage-reared birds (3). However, a similar study failed to find differences in escape or tonic immobility responses between floor and cage-reared laying hens housed in cages as adults (15). Other studies that have tested for effects of exposure to varying degrees of environmental complexity confound effects of rearing and housing of adult birds [see Ref. (16, 17)]. To the best of our knowledge, there are no previous studies comparing effects of rearing in a complex aviary system with rearing in a barren cage environment on fear responses in birds housed in the same environment as adults.

There is consensus that an individual’s fearfulness can be quantified by observing its response to novelty (6, 18–20). Novel object tests measure the conflicting motivations to approach and avoid a novel object, as described by Miller’s Model (21, 22). This model states that the animal will approach a novel object up to the point at which the motivation to avoid the stimuli becomes as strong as the motivation to approach it (21–24). The duration of time spent in proximity to a novel object can, therefore, be used to quantify an animal’s fearfulness.

The aim of this study was to test the hypothesis that birds reared in a conventional rearing cage system would be more fearful when exposed to a novel object during the production period than birds reared in a complex aviary system. The hypothesis was tested in a controlled experimental study where laying hens were reared in either enclosed cages or an aviary system and then transferred at 16 weeks of age to the same house containing furnished cages. Fear responses were evaluated at 19 and 21 weeks of age using the duration of time spent close to a novel object in the home cage. The birds used in the present study are identical to those used by Tahamtani et al. (25). The previous article focused on using undisturbed comfort behavior and alertness in response to a novel object as indicators of animal welfare and not specifically as indicators of fearfulness. Production data, mortality, and blood glucose levels were also presented in the previous article. The current study is conceptually unique in focusing on fearfulness as indicated by approach–avoidance behavior.

## Materials and Methods

### General Description of Subjects and Rearing Conditions

As previously mentioned, the subjects and most of the methods in the current study correspond to Tahamtani et al (25). However, the methods and results for approach–avoidance behavior are unique. Non-beak trimmed, female Lohmann Selected Leghorn chickens (*Gallus gallus domesticus*) of ages 0–21 weeks and normal health status were used in this study within a commercial setting. These birds were hatched and reared in one of two rearing treatments: an aviary- or a conventional cage-rearing system. All eggs originated from the same flock and were incubated at the same time by the same hatchery. Birds in the two treatments were provided with the same feed but were housed in different rooms containing either aviaries or rearing cages at the same farm. Rearing cages measured 6050 cm^2^ and contained 17 birds per cage (Housing Type: Big Dutchman Universa), giving a stocking density of 28 birds/m^2^. The flooring in these cages was wire, and no bedding was provided. The density of birds in the aviary-rearing system (Housing Type: Big Dutchman Natura Rearing) was 24 birds/m^2^. The bedding on the floor of the house was sawdust (small dimension wood shavings). Pullets were provided with *ad libitum* access to feed using a chain dispersal system. The feed type was conventional pullet feed produced and sold by Felleskjøpet, Norway. The diets used were “oppdrett 1” for 0- to 7-week-old birds and “oppdrett 2” for 8- to 17-week-old birds. The nutritional content is optimized for layers of this age according to recommendations by Lohmann (26).

At 16 weeks, the birds from both housing systems were transported to a single farm. The housing at the farm was furnished cages (Housing Type: AVIPLUS, Big Dutchman, designed for housing 10 hens according to EU requirements), measuring 63 cm × 120 cm (7560 cm^2^) and containing between eight and nine birds per cage according to Norwegian legislation. A total of 7500 birds, half of which came from each rearing treatment, were included in the study. The composition of a group was not mixed, cages either contained birds reared in conventional rearing cages or birds reared in the aviary system. The furnished cages included access to dustbathing substrate (a small amount of crushed feed in a 1200 cm^2^, oblong litter bath), a nest box, and two perches. The cages were tiered within the house creating three levels of cages, and arranged in four rows. Each row either contained aviary- or cage-reared birds, allowing birds from different rearing treatments to see one another across an adjacent aisle. The farm operated on a light cycle that was altered according to recommendations by Lohmann (26). During the period of behavioral observations, the light in the chicken house turned on at 07:00 and turned off at 16:00. Feed was provided *ad libitum* using a chain dispersal system in a feeding trough at the front of the cage and water was provided *ad libitum* by nipple drinkers (two per cage).

### Data Collection

The flock at the production farm was visited on two separate occasions during the laying period, once at 19 weeks and again at 21 weeks. Both visits involved the collection of video footage from a selection of cages. A total of 50 furnished cages were recorded at 19 weeks of age, of which 28 contained aviary-reared birds and 22 contained cage-reared birds (see Table 1). At 21 weeks of age, a total of 48 furnished cages were filmed, of which 24 contained aviary-reared birds and 24 contained cage-reared birds. The videos were collected on two consecutive days between the times of 09:00 and 14:00 (see Table 1). The number of cages per tier at the different ages is shown in Table 1. Cage was used as the statistical unit. Cages were selected to represent all areas of the house (row and tier). Different cages were filmed on each farm visit to avoid effects of the first observation upon the second. Two cages from each treatment were filmed concurrently to balance the treatments in case of time effects. After recording had begun, the researcher left the house. Hand-held cameras (Everio, JVC) mounted on tripods were set up, so that the frontal aspect of the cage was filmed. Ten minutes after filming was started, a researcher returned to add the novel objects to the cages. The novel objects used were empty plastic bottles, hung with a wire attachment on the front bars of the cage so that the bottle was just inside the cage approximately 10 cm from its right boundary. The right side of the cage in front of which the novel object was placed contained a nest box, the roof which was the litter area. The researcher then left the room containing the birds and recording continued for a further 10 min. Subsequently, the researchers returned to remove the novel objects and the cameras and assembled them in a different location within the house. Footage collection continued in this manner until the required number of cages was filmed.

**Table 1:** **Overview of data showing the number of cages per treatment per test day and number of cages per treatment per tier at 19 and 21 weeks of age**.

Age	19 weeks				21 weeks			
Test day	Day 1	Day 2	Total		Day 1	Day 2	Total	
Aviary reared	15	13	28		12	12	24	
Cage reared	10	12	22		12	12	24	
Total	25	25	50		24	24	48	

**Age**	**19 weeks**				**21 weeks**			
**Height of cage**	**Tier 1**	**Tier 2**	**Tier 3**	**Total**	**Tier 1**	**Tier 2**	**Tier 3**	**Total**

Aviary reared	8	10	10	28	6	5	13	24
Cage reared	8	6	8	22	5	12	7	24
Total	16	16	18	50	11	17	20	48

#### Novel Object Test

Observer XT 7.0 software (Noldus Information Technology, Wageningen, The Netherlands) was used for behavioral analysis of the footage. The behavioral analysis was conducted on the basis of video recordings by a single researcher who was blind to the rearing background of the birds. When this analysis was started, the cage was divided into four equal zones of increasing distance to the novel object from right to left, using marks on the screen (zone 1 contained the novel object; zone 4 was furthest away from the novel object). Observations commenced 1 min after placement of the novel object into the home cage and measured the duration of time a focal bird spent in the different zones after introducing the novel object. One bird per cage was used. The observation was subsequently continued for 8 min. Before beginning the observation at the time of video-based analysis, a focal subject was selected in the following manner: the video was paused at the start of the observation. Chickens were numbered from left to right, and a bird selected randomly. In the event of the focal subject’s movement out of view of the camera, the protocol for reselection was to observe the bird at the closest proximity and in front of the previous focal bird (closest to the front of the cage), to avoid influencing the duration of occupation in any given zone. Behaviors were coded in such a way that any one code represented the zone of occupation (proximity to novel object). Behavior was recorded continuously, and the durations of time spent in each zone were mutually exclusive.

### Statistical Analysis

The ANOVA model that was used included the factors rearing treatment, tier (top, middle, or bottom), and the interaction between treatment and tier. Day was initially included in the model as a fixed factor but was removed from the model as it did not have any effect. All factors were fixed. Results for ANOVA are presented as *F* values and *p* values. Means and SD are presented for the raw, untransformed data. The duration of times spent in the 1/4, 1/2, and 3/4 of the cage closest to or containing the novel object were used as indicating proximity to the novel object. If needed, these variables were Box–Cox transformed to meet the assumptions of a GLM. Data for the two ages were analyzed separately. All statistical analysis was performed using JMP version 11.0 (SAS Institute Inc., NC, USA).

### Ethical Statement

After reading a detailed formal application for permission to perform this field study (application ID 3868) the Animal Research Authorities (“Forsøksdyrutvalget,” Norwegian Food Authority, Norwegian Government) stated that no specific permission was needed for the activities described in this study. The rearer had previously received permission from the Norwegian Food Authority to rear birds in traditional rearing cages. Following the study, the birds continued to be housed for egg production purposes until their euthanasia at 76 weeks of age. The study did not involve endangered or protected species.

## Results

Upon introduction of the novel object, birds typically vocalized and fled to the opposite end of the cage, but these responses were not scored systematically or quantified.

### Data from Visit at 19 Weeks of Age

#### Duration of Time Spent in Zone 1

For the duration of time spent in zone 1, in which the novel object was situated, there was no effect of treatment (*F*_1,44_ = 2.6227; *p* = 0.1125), tier (*F*_2,44_ = 0.3290; *p* = 0.7214), or the interaction between them (*F*_2,44_ = 0.5828; *p* = 0.5626).

#### Duration of Time Spent in Zone 1–2

When combining the time spent in zone 1 and 2, the aviary-reared birds tended to spend more time closer to the novel object compared to the cage-reared birds (*F*_1,44_ = 3.0103; *p* = 0.0897; aviary-reared: 248.071 ± 137.87 (mean ± SD) s; cage-reared: 170.045 ± 147.285 s). The tier (*F*_2,44_ = 0.8479; *p* = 0.4352) and the interaction between tier and treatment had no effect (*F*_2,44_ = 1.8932; *p* = 0.1627).

#### Duration of Time Spent in Zone 1–3

The aviary-reared birds spent more time close to the novel object compared to the cage-reared birds (*F*_1,44_ = 5.6105; *p* = 0.0223; aviary-reared: 405.857 ± 89.361 s; cage-reared 340.273 ± 112.877 s). The tier did not affect the amount of time spent close to the novel object (*F*_2,44_ = 0.3187; *p* = 0.7287). The duration of time close to the novel object had a tendency to be influenced by the interaction between treatment and tier (*F*_2,44_ = 2.8072; *p* = 0.0712). Aviary-reared birds in furnished cages at the top tier tended to spend more time close to the object compared to cage-reared birds housed on the lowest tier.

### Data from Visit at 21 Weeks of Age

#### Duration of Time Spent in Zone 1

The aviary-reared birds spent more time close to the novel object compared to the cage-reared birds (*F*_1,44_ = 5.2791; *p* = 0.0267; aviary-reared: 48.083 ± 52.531 s; cage-reared 46.375 ± 88.763 s). Tier affected time spent in zone 1 (*F*_2,44_ = 4.3217; *p* = 0.0196), with birds from the middle tier spending more time (79.176 ± 98.420 s) close to the novel object than birds from the bottom tier (33.273 ± 54.019 s) and the top tier (27.75 ± 41.974 s). There was no interaction between treatment and tier (*F*_2,44_ = 0.3753; *p* = 0.6893).

#### Duration of Time Spent in Zone 1–2

When combining the time spent in zone 1 and 2, there was no effect of treatment (*F*_1,44_ = 0.0005; *p* = 0.9831), tier (*F*_2,44_ = 2.1018; *p* = 0.1349), or the interaction between them (*F*_2,44_ = 1.1296; *p* = 0.3328).

#### Duration of Time Spent in Zone 1–3

When combining the time spent in zone 1–3, there was no effect of treatment (*F*_1,44_ = 0.0595; *p* = 0.8084), tier (*F*_2,44_ = 0.2979; *p* = 0.7439), or the interaction between them (*F*_2,44_ = 0.1847; *p* = 0.8320).

## Discussion

This study tested the hypothesis that birds reared in a cage system are more fearful, when exposed to a novel object during the production period, than birds reared in an aviary system. This was supported by results that suggest rearing in a relatively complex aviary system reduces fearfulness in laying hens compared to rearing in a barren cage environment. The observation that aviary-reared birds had a greater duration of time spent in the 3/4 of the cage closest to the novel object at 19 weeks of age and a greater duration of time spent in the 1/4 of the cage closest to the novel object at 21 weeks indicates lower fearfulness in aviary-reared than in cage-reared birds. The treatment effects at the two ages were thus dependent on the definition of proximity to the novel object that was used, suggesting that *a priori* definitions of approach and avoidance may confer disadvantages. In addition to effects of rearing, birds from the middle tier spent more time in the 1/4 of the cage closest to the novel object at 21 weeks of age than birds housed at the bottom or top tiers. The effect of tier may result from differences in the degree of exposure to caretakers as birds in the second tier have the closest proximity to humans during daily inspections. If so, the effect on the response to the novel object may also suggest some generalization of responses from humans to novelty.

Some studies have aimed at testing for effects of rearing conditions on later fear responses in adult laying hens. These are, however, difficult to compare to the present study because adult birds were not transferred to the same type of housing conditions after the rearing period [see Ref. (16, 17)]. This means that birds’ fear reactions may be influenced by rearing but are also likely to be a product of the environment in which they are housed during the time they were observed and tested. To the authors’ knowledge, no previous studies have used an experimental design that does not confound effects of rearing conditions with the housing of adult birds. Furthermore, none of the previous studies compared the effects of rearing in aviaries and cages. Several previous studies do, however, compare rearing in barren cages on wire to rearing on more complex substrates, such as sand, straw (14), or standard litter (3, 15). For the sake of comparison, one can arguably consider environments containing sand, straw, and other substrates to represent a higher degree of environmental complexity than barren cages with a wire floor. If one accepts this premise, increasing environmental complexity during rearing increases active reactions to handling and human presence (reduces the duration of tonic immobility) in birds whether they are housed in a floor system (14) or cages as adults [increased expression of flighty responses (3)]. The present study contributes new knowledge by showing that rearing in a more complex environment reduces the birds’ avoidance of novel, fear-inducing stimuli. Because avoidance is one of the most fundamental characteristics of fear responses, this study is the first to indicate that exposure to increased environmental complexity during rearing reduces fearfulness in adult laying hens at three and five weeks following transfer to a furnished cage system.

Reduced approach and increased avoidance tendencies lie at the core of most operational definitions of increased fearfulness. There are, however, other factors that may also influence fear responses in the present study. On the introduction of the novel object test, a bird could start in the area farthest from the novel object or closest to the novel object and simply stay there if it was unresponsive and inactive. A responsive but inactive bird initially close to the novel object could flee to the area farthest from the object and then stay there. A responsive and active bird could flee to the area farthest from the object and but then continue to move around through the area close to the object afterwards. Also, we cannot exclude the possibility that group dynamics influenced individual fear responses. In the present study, it is not possible to disentangle these potential interacting effects related to responsiveness, activity, and group dynamics. They could all theoretically be confounded with fearfulness and are likely to contribute to residual variation in the data.

The study by Tahamtani et al. (25), using birds that were identical to those used in the current study, indicated that aviary-rearing resulted in birds that displayed more comfort behavior at 19 weeks of age but had higher mortality throughout the production period. Higher expression of comfort behavior in aviary-reared birds suggests that they had better welfare, whereas the greater mortality throughout production suggests the opposite. The findings in the present study correspond well to the previously documented effect on comfort behavior but stand in contrast to the effect on mortality. Our results contradict the interpretation of Anderson and Adams (3), who suggested that birds showing active escape attempts (floor-reared birds) are more fearful than birds showing more passive responses (cage-reared birds). However, these researchers used test conditions (a human stimulus) and categorized behavior (from “calm, no nervous or evasive action” category 0 to “extreme escape and avoidance behavior” category 4) using an approach that was rather different than we used in the current study. Because observations were based on a combination of qualitative measures, they are difficult to interpret. Furthermore, results by Brantsæter et al. (27) indicate that flight responses when suddenly exposed to novel stimuli may not be related to fearfulness in laying hens but are more likely to reflect coping style.

Because observations were only carried out at 19 and 21 weeks of age but not later, the present study may not have clear implications for bird welfare in the long term. However, the treatment effect found at 19 weeks of age corresponds to a time at which birds are dealing with hormonal changes associated with the onset of lay [see Ref. (28)]. The positive effect of rearing in aviaries could, therefore, be important from an animal welfare perspective. The current study focused on the early production phase soon after transfer to the production facility. Future research could test whether the effect of rearing persists to later stages of development, especially given the previously reported negative effects of aviary rearing on mortality throughout the production phase (25). Farmers are unlikely to use aviary rearing as a method of reducing fearfulness in laying hens specifically, especially if they should later be used for producing in cages. It would, therefore, be useful to test effects of measures for reducing fearfulness that could be used in practice for conventional laying hens.

## Author Contributions

MB analyzed the data and wrote the first draft of the article; FT analyzed the data; RM collected data; TH collected data; RO scored behavior; CN contributed to conception and design of the study; AJ led the project, participated in conception and design of the study and collected the data. All authors contributed in writing the manuscript and approved the final version.

## Conflict of Interest Statement

No conflicts of interest exist in regards to this study. The funding organizations, the Foundation for Research Levy on Agricultural Products (FFL), the Agricultural Agreement Research Fund (JA), and Animalia (Norwegian Meat and Poultry Research Centre) finance applied agricultural research in collaboration with the private and public sectors. These parties’ sole interest in the present study was to support publication of unbiased results in order to provide advice to poultry rearers.
